# Abnormal Lipid Profile in Fast-Growing Broilers With Spontaneous Femoral Head Necrosis

**DOI:** 10.3389/fphys.2021.685968

**Published:** 2021-06-14

**Authors:** Rubin Fan, Kangping Liu, Zhenlei Zhou

**Affiliations:** Department of Veterinary Clinical Science, College of Veterinary Medicine, Nanjing Agricultural University, Nanjing, China

**Keywords:** femoral head necrosis, bone metabolism, broiler, hyperlipidemia, lipid metabolism disorder

## Abstract

This study investigated lipid metabolism in broilers with spontaneous femoral head necrosis (FHN) by determining the levels of markers of the blood biochemistry and bone metabolism. The birds were divided into a normal group and FHN group according to the femoral head scores of 3-, 4-, and 5-week-old chickens with FHN, and a comparative study was conducted. The study showed that spontaneous FHN broilers had a lipid metabolism disorder, hyperlipidemia, and an accumulation of lipid droplets in the femur. In addition, there were significant changes in the bone parameters and blood bone biochemistry markers, and the expression of genes related to lipid metabolism in the femoral head was also significantly increased. Therefore, FHN may result from dyslipidemia, which affects the bone growth and development of broilers.

## Introduction

In the past few decades, substantial progress has been made in improving the feed efficiency and growth rate in fast-growing broilers, and the growth rate and body weight at market age have increased almost threefold (Havenstein et al., 2003). However, the heavier body weight also exerts negative effects on body health, and metabolic problems are promoted, such as pulmonary hypertension, fatty liver syndrome, lameness, and skeletal problems (Packialakshmi et al., 2015b). Femoral head necrosis (FHN) is one of the most common leg problems, and it leads to lameness and affects the growth and development of broilers. According to investigations and studies, at a high stock density, the incidence of leg problems is about 2%, and the detection rate of FHN is as high as 13.33% (Applegate and Lilburn, 2002; Li et al., 2015), which often affects broilers at 5 to 6 weeks of age. FHN is often characterized by the squatting position and rarely standing and walking, and severely affected broilers even limp. Due to the limited access to feed, FHN not only drastically reduces the production performance of broiler chickens, giving rise to a poor bird welfare, but also causes considerable economic losses (Julian, 2005).

A disorder of lipid metabolism is considered to be one of the main factors in FHN-affected broilers (Brewer et al., 1972; Yamamoto et al., 1997). The pathogenesis of FHN is often accompanied by the occurrence of hyperlipidemia (Yu et al., 2016; Liu et al., 2021). However, the influences of lipid metabolism disorder are not clearly understood in FHN broilers. It is believed that if blood lipids are abnormal, the levels of, for example, triglycerides (TG), lipoprotein (LDL), and total cholesterol (TC) increase significantly, the high blood viscosity lowers the blood flow, and, as a result, fat emboli in blood vessels develop (Durairaj et al., 2009; Packialakshmi et al., 2015a; Wang et al., 2018; Guo et al., 2019). Then, the intramedullary microcirculation problem causes tissue hypoxia, high intraosseous pressure, and deficient nutrient transport, which ultimately promotes bone cell necrosis (Lee et al., 2006). In addition, neutral fatty acids can result in capillary intima falling off, vascular wall edema, intravascular congestion, and the aggravation of the femoral head ischemia (Teitelbaum, 2000).

Bone is a dynamic endocrine organ that regulates its own and even the whole body’s steady-state balance (Alekos et al., 2020). Bone modeling and remodeling are strictly controlled by many factors, including lipids. The lipids in bones are generally considered to exist only in the bone marrow, and the mineralized bone tissue itself contains a small amount of lipids, which may play an important role in bone physiology. Among the total fatty acids, for chicken, the bones mainly contain oleic acid (18:1n-9) (35–45%) and palmitic acid (16:0) (25%) [% of the total fatty acids (Wolinsky and Guggenheim, 1970)], which is similar to the distribution of fatty acids in human bones (Dirksen and Marinetti, 1970). Among the bone components, chicken cortical bone contains a higher fatty acid content than cancellous bone, which allows the intensity of metabolic activity to be maintained (Dolegowska et al., 2006; Liu et al., 2021). In addition, the utilization of fatty acids in bone is comparable to that in tissues with more a classic fatty acid metabolism (Alekos et al., 2020). Increasing evidence shows that there is a close correlation between bone fat and many bone diseases (Bermeo et al., 2014). In patients with osteoporosis, abnormal blood lipid levels are associated with a decreased bone density and bone loss (Nuzzo et al., 2009; Chen et al., 2016; Saoji et al., 2018; Chi et al., 2019). In addition to hyperlipidemia, mice receiving a high-fat diet treatment also showed trabecular bone destruction and a decreased bone strength (Chen et al., 2016).

Despite extensive research, the biological mechanisms underlying the relationship between FHN and lipid metabolism disorders in broilers and other animals is an issue that is worth investigating. The aim of this study was to determine how spontaneous FHN is influenced by lipid metabolism disorders in broilers by analyzing blood lipid metabolism markers and the expression of related genes in the femoral head and bone parameters.

## Materials and Methods

### Sample Collection

The broilers (Gallus gallus, AA broilers) of both sexes were sampled from a chicken farm in Jiangsu province. The birds were fed a two-phase commercial diet *ad libitum*: a starter ration (21.00% crude protein, 1.00% Ca, 0.52% total P, and 0.45% methionine) from 0 to 21 days and a grower ratio (19.00% crude protein, 0.95% Ca, 0.47% total P, 0.38% methionine) from 22 to 35 days. At the ages of 3, 4, and 5 weeks, eight normal broilers and eight FHN-affected broilers were selected for the collection of serum, plasma, and tissue samples. After the body weight was recorded, blood samples were collected from the wing vein and centrifuged at 4,000 *g* for 15 min. The serum and plasma were collected and then stored at −20°C for analysis. The liver samples from the broilers at the age of 5 weeks were collected immediately, rinsed with normal saline, and fixed in 4% formaldehyde. Both sides of the femur and tibia from the normal and FHN-affected birds were harvested and maintained at −20°C for the determination of the bone density and biomechanics. The femoral head was cut along the sagittal plane and rinsed with normal saline. One half was cleaned with PBS and fixed in 4% paraformaldehyde at 4°C, and the other half was subjected to DEPC and then stored in liquid nitrogen. According to the FHN diagnosis standard, the broilers were divided into two groups: a normal group and FHN-affected group.

### Biochemistry

The levels of serum alkaline phosphatase(ALP), Ca, P, triglycerides (TG), total cholesterol (TC), high-density lipoproteins (HDL), and low-density lipoproteins (LDL) were detected using a BS-300 automatic biochemical detector (Mindray Biomedical Electronics Co., Ltd.). Each sample was measured in triplicate.

### ELISA Assay

Two kinds of indicants in the plasma were measured using a chicken-specific ELISA kit. Seven indicators related to lipid metabolism were determined, including acetyl-CoA carboxylase (ACC), fatty acid synthase (FAS), lipoprotein lipase (LPL), adipose triglyceride lipase (ATGL), hormone-sensitive lipase (HSL), peroxisome proliferator-activated receptor (PPARγ), and free fatty acid (FFA). Two indicators of bone metabolism were analyzed, including bone alkaline phosphatase (BALP) and tartrate resistant acid phosphatase(TRACP-5b. Each sample was measured in triplicate.

### HE Staining

The femoral head was fixed in 4% PFA, rinsed overnight with tap water, and decalcified with 10% EDTA for 2 weeks. After the treatment, the bone tissue and the PFA-fixed liver tissue were dehydrated with ethanol, cleared with xylene, and embedded in paraffin. Hematoxylin-eosin (HE) staining was performed to observe the degree of lesion changes. The area of lipid droplets in the liver and femoral head were analyzed using ImageJ (Huang et al., 2018; Jahejo et al., 2020).

### Bone Parameters

The bone mineral density (BMD) of the tibia and femur were measured using a dual energy X-ray bone densitometer (MEDIKORS, Gyeonggi, South Korea). The test mode was set with a high energy parameter of 80 kVp/1.0 mA and a low energy parameter of 55 kVp/1.25 mA. The InAlyzer 1.0 image processing system was used to analyze and process the captured pictures and measure and record the length of the femur and tibia and the density of the midsection (Li et al., 2015).

The strength of the tibia and femur was determined using a three-point bending test. The tibia and femur were placed on the working platform of a universal material testing machine (LR10K Plus, Lloyd Instruments Ltd., United Kingdom), with a known span. The following parameters were set: preload: 5 N; preload speed: 15 mm/min; and the test was stopped when the bone was fractured. The bone strength curve was obtained by NEXYGEN Plus software. The highest point of the curve was the bone strength value (N).

### Total RNA Extraction and RT q-PCR

The femoral head samples in broilers at the age of 5 weeks were pulverized in a low-temperature environment and treated with trizol (Nanjing Angel Gene Biotechnology Co., Ltd., Nanjing) to extract the total RNA. The use of HiScript II QRT SuperMix for qPCR (+ gDNA wiper; Zazyme, Nanjing, China) resulted in reverse transcriptional synthesis of complementary DNA. Using the ABI PRISM 7300 HT sequence detection system (Application Biossystems, Inc., Foster City, CA, United States), SYBR Green PCR technology was used to detect the expression of lipid metabolism-related genes. All PCR operations were performed in triplicate. The selected genes and the primer sequences for the corresponding genes are shown in Table 1. Housekeeper GAPDH was used as an internal standard for the quantity and quality of cDNA.

**TABLE 1:** Sequences of primers used to express specific mRNAs by RT q-PCR.

Target gene	Primer sequence (5′-3′)
ACSL1	F:TGGAACGTGGCAAGAAGTGT
	R: CCCTTGGGGTTTCCTGTTGT
ACC	F: GCTTCCCATTTGCCGTCCTA
	R:GCCATTCTCACCACCTGATTACTG
FAS	F: TTTGGTGGTTCGAGGTGGTA
	R: CAAAGGTTGTATTTCGGGAGC
CPT1	F: TAGAGGGCGTGGACCAATAA
	R: TGGGATGCGGGAGGTATT
ATGL	F: TCCTAGGGGCCTACCACATC
	R: CCAGGAACCTCTTTCGTGCT
PPARγ	F: GTGCAATCAAAATGGAGCC
	R: CTTACAACCTTCACATGCAT
LRP1	F: CTCTGTGGATTGGGTTTCC
	R: ACCAGGCAGTGGGGTTTA
GAPDH	F: GAACATCATCCCAGCGTCCA
	R: CGGCAGGTCAGGTCAACAAC
APOB	F: GCAGCCTATGGAACAGA
	R: TAGTGGAACGCAGAGCA

### Statistical Analysis

The statistical analysis was conducted using SPSS 17.0 for windows. The data were expressed as the mean ± SEM, and the differences between groups were determined using one-way analysis of variance (ANOVA, SNK). Significant differences were accepted at two levels: P < 0.05 (significant) and P < 0.01 (extremely significant).

## Results

### FHN Affected the Bone Growth in Broilers, Especially That of the Femur

**FIGURE 1:**
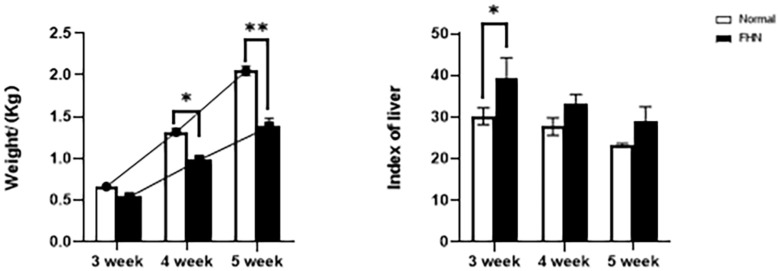
The weight and liver coefficient changes for 3∼5 weeks of FHN. ^∗^indicates a significant difference (*P* < 0.05). ^∗∗^indicates an extremely significant difference (*P* < 0.01).

**FIGURE 2:**
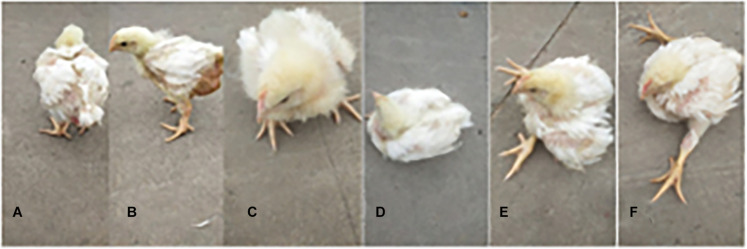
The varying degrees of gait changes in broilers with FHN. Gait score of 0 **(A)** The bird walked normally, with no detectable abnormality; Gait score of 1 **(B)** The bird had a slight defect, which was difficult to define precisely; Gait score of 2 **(C)** The bird had a definite and identifiable defect in its gait; Gait score of 3 **(D)** The bird had an obvious gait defect, which affected its ability to move about; Gait score of 4 **(E)** The bird had a severe gait defect; Gait score of 5 **(F)** The bird was incapable of sustained walking on its feet.

**FIGURE 3:**
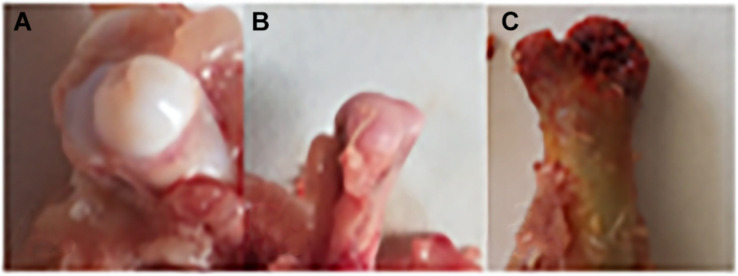
Clinical and anatomical manifestations of FHN broilers. **(A)** Normal broilers; **(B,C)** FHN-affected broilers. Panel **(B)** shows a cartilage loss, and the subchondral bone had ivory-like changes; Panel **(C)** indicates a cartilage separation, and the epiphysis is broken.

The chicken farm has a total of 252,000 chickens, with a mortality rate of 3.80% and a culling rate of 1.15%. Of the 80 lame chickens examined, 48 had FHN, and their gait scores (Kestin et al., 1992) were point 4.8 for normal broilers and 8 for FHN-affected chickens when analyzed at 3, 4, and 5 weeks. The body weight of the normal broiler chickens increased from the 3th to the 5th weeks, but the body weight of the chickens with FHN decreased significantly in the 4th and 5th weeks (*P* < 0.05). The body weight of the FHN birds (1.227 ± 0.103 kg) was significantly lighter than that of the normal ones (2.050 ± 0.053 kg) (*P* < 0.01) in the 5th week (Figure 1). In addition, most broilers suffering from FHN showed an abnormal gait. The varying degrees of gait changes in the broilers with FHN are shown in Figure 2. FHN in broilers was defined as a separation of the femoral head cartilage from the underlying growth plate, damage to the growth plate, or even a breakage of the epiphysis. Figure 3 shows the apparent changes of the FHN-affected broilers.

**FIGURE 4:**
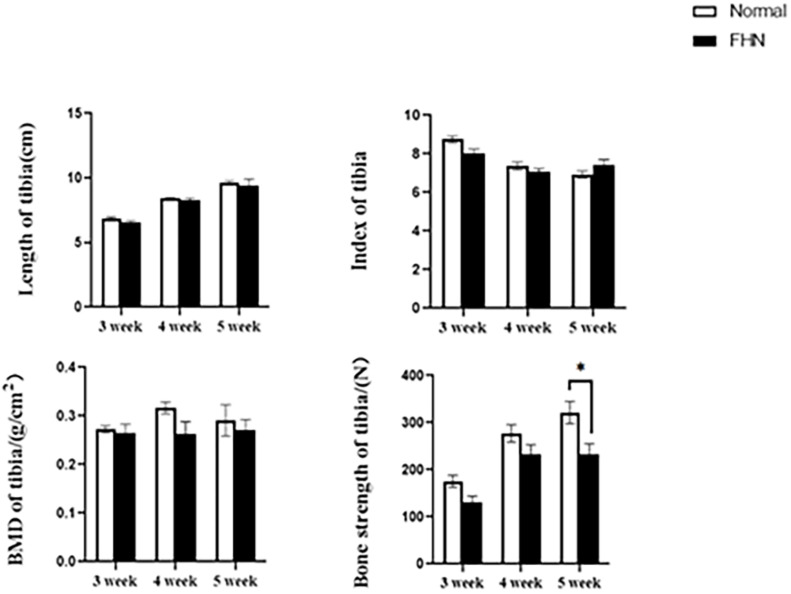
Tibia parameters in broilers. ^∗^indicates a significant difference (*P* < 0.05).

**FIGURE 5:**
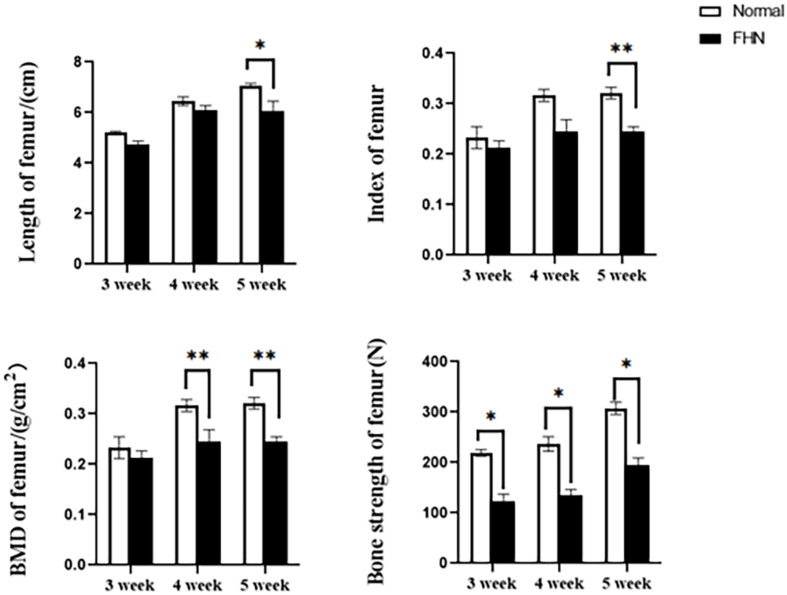
Femur parameters in broilers. ^∗^indicates a significant difference (*P* < 0.05). ^∗∗^indicates an extremely significant difference (*P* < 0.01).

**FIGURE 6:**
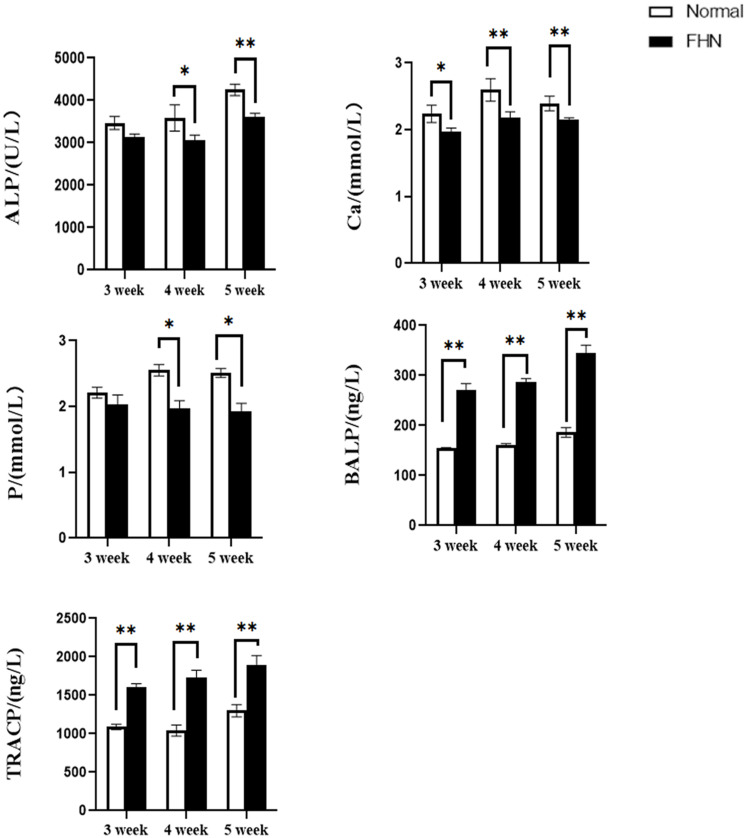
The changes of serum biochemical indicators and bone metabolism. ^∗^indicates a significant difference (*P* < 0.05). ^∗∗^indicates an extremely significant difference (*P* < 0.01).

The bone length, bone index, bone strength, and bone density of the tibia did not differ significantly between the normal and the FHN-affected group, except for the tibia bone strength in the 5th week, and the difference in the performance of the bone index on the femur was very obvious. The length and bone index of the femur in the FHN group in the 5th week were significantly lower than those in the normal group (*P* < 0.05). The bone density of the femur was also significantly decreased in the 4th and 5th weeks in the FHN group. At the same time, the bone strength in the FHN group decreased significantly in 3∼5 weeks (*P* < 0.05). The results show that FHN affected the bone density and strength of long bones, especially the femur (Figures 4, 5). The levels of ALP, Ca, and P in the FHN group were lower than those in the normal group in the 4th and 5th weeks (*P* < 0.05). In addition, the BALP and TRACP activities in the FHN group were elevated (*P* < 0.05), indicating that the bone formation and bone resorption activities were enhanced in the pathogenesis of FHN (Figure 6).

### Lipid Metabolism Disorder Appeared in FHN

**FIGURE 7:**
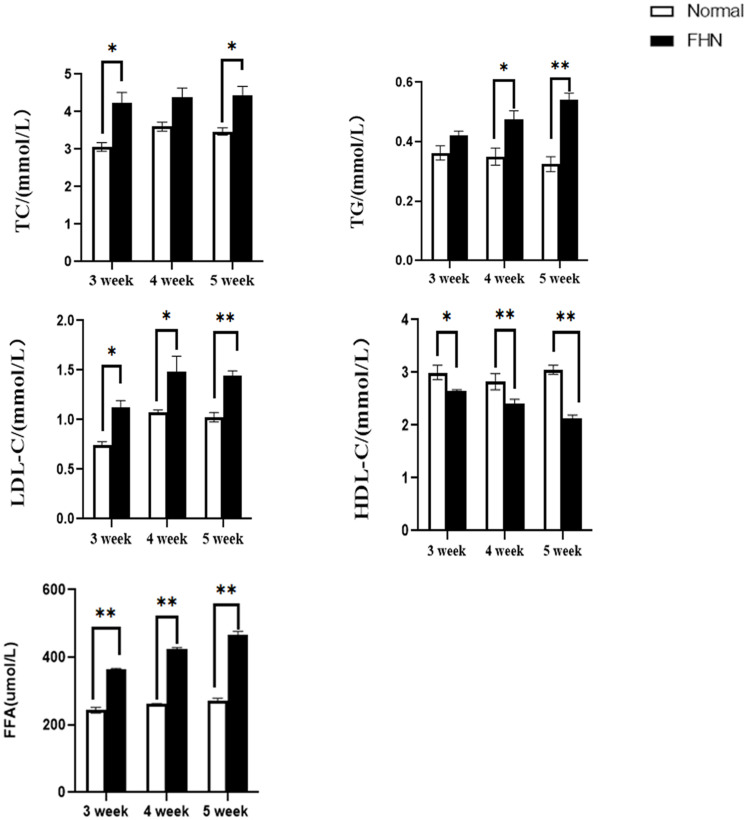
The changes in serum biochemical indicators regarding lipid metabolism. ^∗^indicates a significant difference (*P* < 0.05). ^∗∗^indicates an extremely significant difference (*P* < 0.01).

Compared with normal broilers, a higher TC, TG, and LDL-C were detected in the FHN broilers, and the HDL-C was lower, leading to an elevated FFA (Figure 7).

**FIGURE 8:**
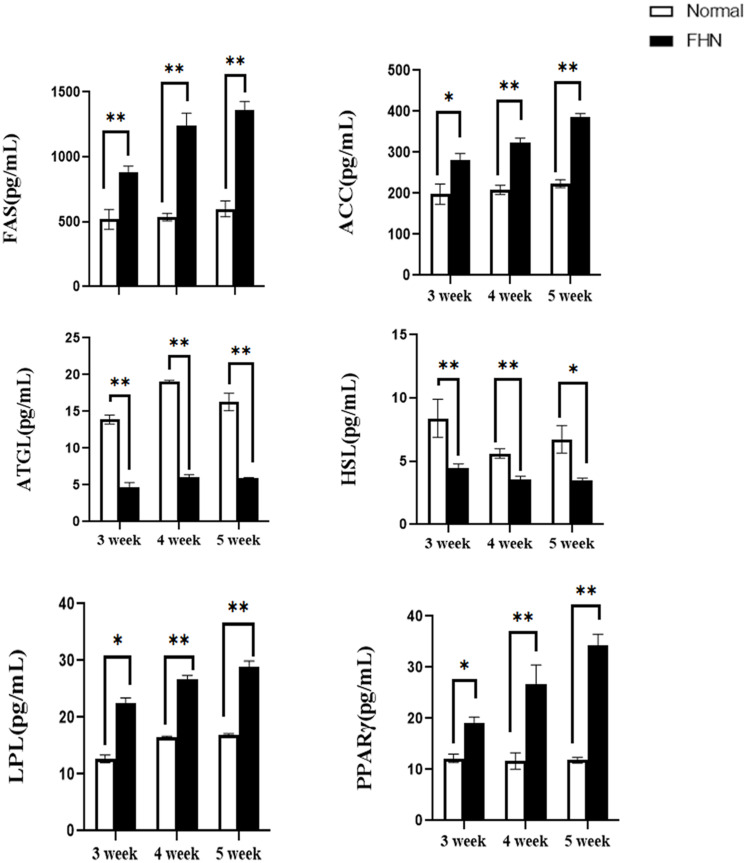
The changes in serum lipid metabolism-related enzymes and markers. ^∗^indicates a significant difference (*P* < 0.05). ^∗∗^indicates an extremely significant difference (*P* < 0.01).

Lipid metabolism-related enzymes and markers were detected using an ELISA. Two key rate-limiting enzymes affecting FAS and ACC were significantly increased in the FHN group, and the two enzymes involved in ATGL and HSL were significantly decreased. Compared with the normal group, the fat synthesis and decomposition of the FHN group were increased, which also caused an accumulation of fat. The increase in LPL and PPARγ in the FHN group also indicated this trend, as shown in Figure 8.

**FIGURE 9:**
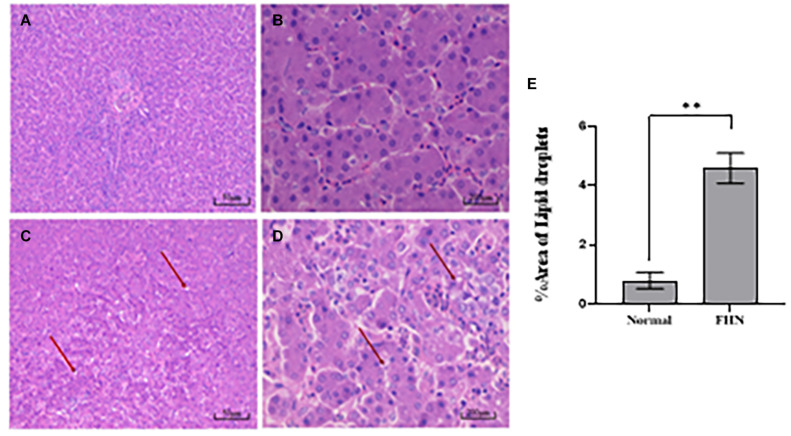
HE staining of the liver in broilers at the age of 5 weeks. Normal broilers: **(A,B)**; FHN broilers: **(C,D)**; analysis of the area of lipid droplets in the liver **(E)**. The red arrow points to the site where vacuolar degeneration occurred. ^∗∗^indicates an extremely significant difference (*P* < 0.01).

**FIGURE 10:**
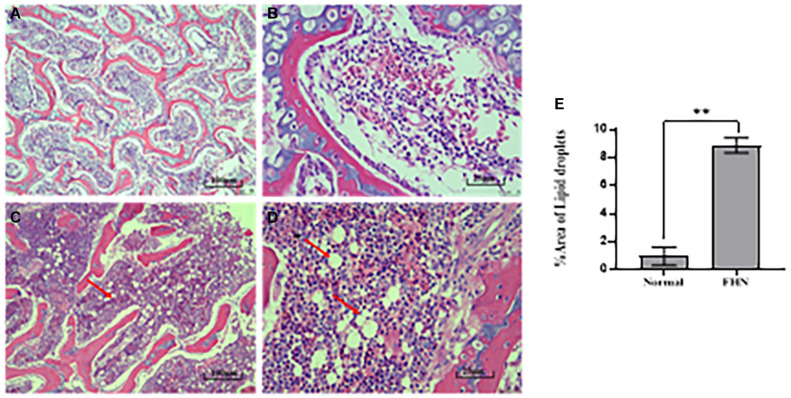
HE staining of the femoral head in the broilers at the age of 5 weeks. Normal broilers: **(A,B)**; FHN broilers: **(C,D)**; analysis of the area of lipid droplets in the femoral head **(E)**. The red arrow points to the site where vacuolar degeneration occurred. ^∗∗^indicates an extremely significant difference (*P* < 0.01).

**FIGURE 11:**
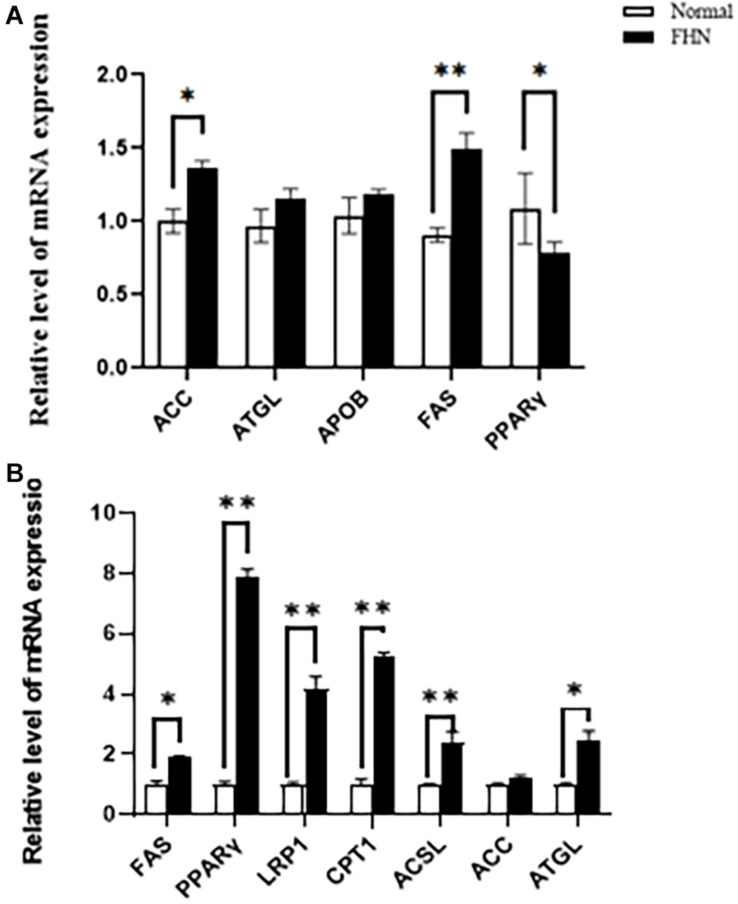
The changes of the mRNA expression of the target genes related to lipid metabolism in the liver **(A)** and femoral head **(B)** at the age of 5 weeks. ^∗^indicates a significant difference (*P* < 0.05). ^∗∗^indicates an extremely significant difference (*P* < 0.01).

HE staining (Figure 9) showed that the liver cells in the control broilers at the age of 5 weeks had a normal morphology and complete structure, while in the broilers affected by FHN, round cavities of different sizes appeared, indicating that the liver structure in the FHN birds was damaged to varying degrees. The liver of the FHN-affected broilers was likely to have steatosis. In addition, HE staining (Figure 10) reflected the presence of lipid droplets of different sizes in the subchondral bone in the FHN birds, indicating that the femoral head was infiltrated with fat when necrosis occurred. In addition, the gene expression related to the fatty acid synthesis pathway in the liver was detected, including an increase in ACC and FAS and a decrease in PPARγ, which resulted in lipid deposition during liver metabolism (Figure 11A).

### Expression of Lipid-Related Genes

Figure 11B shows the mRNA expression of some genes related to lipid metabolism in the subchondral bone of the femoral head in the broilers at the age of 5 weeks. Compared with the normal group, the fat mobilization genes, including CPT1, ACSL, and ATGL, were significantly promoted in the FHN birds, and the genes related to fat synthesis were also significantly increased, including FAS, PPARγ, and LRP1.

## Discussion

Recent studies have shown that with the deepening of lipid metabolism disorders, abnormal bone metabolism, osteoarthritis, osteoporosis, bone loss, and other diseases may occur, and the risk of fractures increases. Gu et al. (2019), Yang et al. (2019) found that there is a negative relationship between fat intake and bone density. Mice fed a high-fat for a long time show a decrease in the amount of osteoid, cancellous bone, and type I collagen. Osteoporosis is also closely related to hyperlipidemia (During, 2020). As the plasma TC concentration increases, the bone density decreases. Clinically, statins are used to antagonize osteoporosis (Chamani et al., 2021). In addition, the inflammatory microenvironment caused by fat has a stimulating effect on the formation of bone cells, releasing a large number of inflammatory factors, aggravating the destruction of cartilage damage and synovial inflammation, and increasing the incidence of arthritis (Pousinis et al., 2020).

Lipid metabolism disorder has been recognized as one of the factors of femoral head necrosis, particularly hormonal femoral head necrosis, which is mainly attributed to hyperlipidemia. Hormonal drugs cause high blood coagulation and low fibrinolysis and thrombosis; impair the blood flow in bone, leading to ischemia, hypoxia, and the necrosis of bone cells; destroy the structure and function of bone tissue; and, finally, result in avascular necrosis of the femoral head (Glueck et al., 2005). Previous studies have found that lipids are distributed in bone marrow and mineralized tissues and may play important roles in regulating the physiological functions of bone fatty acids, cholesterol, phospholipids, and several endogenous metabolites [such as prostaglandins and oxysterols (During et al., 2015)] that play important roles in bone cell survival and function, bone mineralization, and key signaling pathways. Therefore, they can be considered as important regulators of bone homeostasis (Kiyoi, 2018). However, on the other hand, fatty acids have toxic effects and can impair bone health (Innes and Calder, 2018).

Most of the findings still remain restricted to humans and mice. However, in recent years, broilers have been used as a model animal for leg disorder research. This study used naturally affected broilers as a model to study the relationship between lipid metabolism disorders and femoral head necrosis during the pathogenesis of FHN in broilers. In fast-growing broilers, the incidence of FHN is higher at 4∼6 weeks due to the linear increase in body weight in these periods. Compared with the normal broilers, the body weight of the FHN birds significantly decreased because it was difficult for the FHN birds to access feed and water. We found that many of the naturally FHN-affected broilers were unwilling to walk or stand, and there were also a few broilers that looked normal in appearance and gait, but an autopsy showed that these birds were affected by FHN. Therefore, it was a golden standard to diagnose FHN based on pathological changes, clinical signs, and gait observation. In addition, bone quality is an important indicator of bone health and can be assessed through the determination of bone parameters (Fonseca et al., 2014). The bone strength and density of broilers with FHN were significantly decreased. This situation was also directly manifested in the abnormal gait of the FHN chickens, which was prone to fracture during autopsy. Serum ALP, Ca, and P showed a downward trend. The abnormal increase in BALP and TRACP indicated that the balance between bone formation and bone resorption was disturbed. In this work, the occurrence of FHN directly affected the maintenance of long bone volumes in broilers, especially the femur.

Lipid metabolism disorder appeared in broilers with FHN. The indicators of liver function were significantly higher in the FHN-affected birds. Histopathology showed that lipid droplets and steatosis appeared in the liver of the FHN birds. Combined with the manifestations of hyperlipidemia in the plasma, TC, TG, and LDL-C were increased, and HDL-C was decreased, indicating that the broiler chickens with FHN had a lipid metabolism disorder. When FHN developed, the body was in a state of lipid accumulation. The bone itself is equipped with enzymes and receptors for the absorption and utilization of fatty acids. The intake of chylomicrons of bone accounts for 17% of the liver, which is higher than that of other catabolic organs, such as muscle and the heart (Niemeier et al., 2008). Importantly, a femoral shaft rich in osteoblasts/osteocytes has a higher residual uptake of chylomicrons than bone marrow (Bartelt et al., 2017). This shows that the femur can absorb circulating lipoproteins and free fatty acids. We observed lipid droplets in the subchondral bone of the femoral head in the FHN birds. The potential role of bones in fatty acid metabolism is emphasized in the pathogenesis of FHN.

Previously, some people performed gene-chip analysis in hormonal osteonecrosis model rats and found that genes related to fatty acid synthesis in the femoral head were up-regulated (Yue et al., 2018). This finding is similar to our current results. The Acsl1 and CPT1 genes were up-regulated, and the FAS expression was down-regulated. More similarly, the PPAR gene also showed down-regulation, like FHN in the other animals. PPARγ is a widely expressed nuclear transcription factor, which plays an important role in the differentiation of bone marrow mesenchymal stem cells (Kim and Ko, 2014; Yuan et al., 2018). It promotes audiogenic differentiation at the expense of inhibiting osteogenic differentiation (Wang et al., 2017). Most studies reported that the main reason for glucocorticoid-induced FHN is an abnormal audiogenic osteogenic differentiation of bone marrow mesenchymal stem cells, which is manifested by an increased differentiation of adipocytes and inhibition of osteoblast differentiation (Yu et al., 2016). We speculate that the femoral head of FHN broilers may also have a similar abnormal pathological change and an increased fat accumulation in the femoral head. In addition, the expression of the lrp1 gene was up-regulated. It was reported that in the culture of osteoblast models, LRP1 promoted the endocytosis of triglycerides and cholesterol containing chylomicron residues (Frey et al., 2014). The polymorphism of the gene encoding this receptor is related to bone density and to the maintenance of bone stability (Sims et al., 2008; Frey et al., 2014). LRP1 regulates osteoblast and osteoclast activities through the Wnt pathway. The Wnt pathway is the opposite of PPARγ, which also controls the osteogenic-adipogenic differentiation of bone marrow mesenchymal stem cells and promotes the proliferation and differentiation of osteoblasts (Zhang et al., 2015; Chen et al., 2016; Yao et al., 2017). In this work, the osteogenic-adipogenic differentiation of femurs in FHN broilers was disrupted, many fat cells appeared, the fatty acid metabolism increased, and the lipotoxic effect of fatty acids caused the necrosis of bone cells and osteoblasts. However, the reason for the destruction of the balance of osteogenic-adipogenic differentiation was not yet known, and further research in this direction needs to be conducted.

In summary, when femoral head necrosis occurred in fast-growing broilers, it was accompanied by lipid metabolism disorders. The affected femoral head had a lipid accumulation and changes in the expression of lipid metabolism-related genes, which may be involved in the PPAR pathway. A lot of work needs to be conducted to figure out the factors leading to femoral head necrosis in broilers, and the mechanisms of lipid regulation involved in osteocyte function and signal pathways need to be investigated.

## Data Availability Statement

The raw data supporting the conclusions of this article will be made available by the authors, without undue reservation.

## Ethics Statement

The animal study was reviewed and approved by #NJAU-Poult-2020092403, approved on September 24, 2020.

## Author Contributions

RF helped with the experimental design and this writing. KL helped with the sampling and the data analysis. All authors have read and agreed to the published version of the manuscript.

## Conflict of Interest

The authors declare that the research was conducted in the absence of any commercial or financial relationships that could be construed as a potential conflict of interest.
